# Activation of AMPKα2 Is Not Required for Mitochondrial FAT/CD36 Accumulation during Exercise

**DOI:** 10.1371/journal.pone.0126122

**Published:** 2015-05-12

**Authors:** Cynthia Monaco, Jamie Whitfield, Swati S. Jain, Lawrence L. Spriet, Arend Bonen, Graham P. Holloway

**Affiliations:** Department of Human Health and Nutritional Sciences, University of Guelph, Guelph, ON, Canada; INSERM/UMR 1048, FRANCE

## Abstract

Exercise has been shown to induce the translocation of fatty acid translocase (FAT/CD36), a fatty acid transport protein, to both plasma and mitochondrial membranes. While previous studies have examined signals involved in the induction of FAT/CD36 translocation to sarcolemmal membranes, to date the signaling events responsible for FAT/CD36 accumulation on mitochondrial membranes have not been investigated. In the current study muscle contraction rapidly increased FAT/CD36 on plasma membranes (7.5 minutes), while in contrast, FAT/CD36 only increased on mitochondrial membranes after 22.5 minutes of muscle contraction, a response that was exercise-intensity dependent. Considering that previous research has shown that AMP activated protein kinase (AMPK) α2 is not required for FAT/CD36 translocation to the plasma membrane, we investigated whether AMPK α2 signaling is necessary for mitochondrial FAT/CD36 accumulation. Administration of 5-Aminoimidazole-4-carboxamide ribonucleotide (AICAR) induced AMPK phosphorylation, and resulted in FAT/CD36 accumulation on SS mitochondria, suggesting AMPK signaling may mediate this response. However, SS mitochondrial FAT/CD36 increased following acute treadmill running in both wild-type (WT) and AMPKα 2 kinase dead (KD) mice. These data suggest that AMPK signaling is not required for SS mitochondrial FAT/CD36 accumulation. The current data also implicates alternative signaling pathways that are exercise-intensity dependent, as IMF mitochondrial FAT/CD36 content only occurred at a higher power output. Taken altogether the current data suggests that activation of AMPK signaling is sufficient but not required for exercise-induced accumulation in mitochondrial FAT/CD36.

## Introduction

The transport of fatty acids across biological membranes is now recognized to be a protein-mediated process (reviewed in [1]). Three types of sarcolemmal fatty acid transport proteins have been identified in muscle, including plasma membrane fatty acid binding protein (FABPpm) [2], a family of fatty acid transport proteins (FATPs) [3–5], and fatty acid translocase (FAT/CD36) (FAT is the homolog of human CD36) [6]. Of these, FAT/CD36 displays the greatest positive effect on fatty acid transport rates [7]. In addition, ablating FAT/CD36 prevents, while over-expressing FAT/CD36 independently mirrors, typical exercise-training induced increases in skeletal muscle fatty acid oxidation [8]. Therefore, FAT/CD36 is considered integral in regulating fatty acid metabolism and responses to chronic exercise training.

In addition to its influence on chronic exercise-responses, FAT/CD36 regulates plasma membrane fatty acid transport acutely in response to various stresses. For instance, a number of studies utilizing acute muscle contraction [9–12] suggest that FAT/CD36 translocates between an intracellular depot and the plasma membrane, thereby regulating fatty acid import into muscle cells (reviewed in [1]). In addition to these physiological perturbations, 5-Aminoimidazole-4-carboxamide ribonucleotide (AICAR) exposure can induce FAT/CD36 sarcolemmal translocation in skeletal muscle and in cardiac myocytes [11, 13, 14]. While these studies implicate AMP activated protein kinase (AMPK) in the signaling events that regulate plasma membrane FAT/CD36 accumulation, exercise-induced translocation of FAT/CD36 to the plasma membrane is retained in AMPKα2 kinase dead (KD) mice [11], suggesting AMPKα2 signaling is not required for translocation of FAT/CD36 to the sarcolemma.

Unexpectedly, FAT/CD36 has also been reported on mitochondrial membranes from 7 independent laboratories [15–21]. While controversy exists regarding the functional role of mitochondrial FAT/CD36, we have provided evidence to suggest that FAT/CD36 interacts with acyl-CoA synthetase to regulate fatty acid oxidation. Specifically, in FAT/CD36 knock out mice, palmitate, but not palmitoyl-CoA-supported respiration is impaired, while FAT/CD36 colocalizes on the mitochondrial outer member, and not in contact sites [17]. In addition, another laboratory has shown that transfection of FAT/CD36 cDNA into L6E9 myotubes increases mitochondrial palmitate oxidation rates [20], while we have also shown that exercise-induced increases in mitochondrial fatty acid oxidation rates are attenuated in FAT/CD36 null mice [22]. Therefore, increased mitochondrial FAT/CD36 protein is associated with an increase in mitochondrial fatty acid oxidation rates, although the mechanism-of-action remains controversial [19].

Acute exercise causes an increase in mitochondrial FAT/CD36 protein content in mice, rats and humans [15, 23–25]. However, to date studies have not investigated the potential signals responsible for FAT/CD36 accumulation on mitochondrial membranes during exercise, knowledge that may also provide further insight into its functional role within mitochondria. To this end we have investigated the temporal response to muscle contraction-induced FAT/CD36 accumulation, determined if activation of AMPK signaling is involved in mediating these responses, and if exercise intensity influences the magnitude of mitochondrial FAT/CD36 accumulation. We provide evidence that activation of AMPK is sufficient, but not required, for exercise-induced increases in mitochondrial FAT/CD36.

## Methods

### Animals

Male Sprague-Dawley rats (weighing ~300 g) were utilized to determine the temporal response for FAT/CD36 accumulation on sarcolemmal and plasma membranes during muscle contractions as a result of the tissue requirements, however all other experiments were performed in mice. Specifically, 25 week old male wild type mice were used to determine the ability of AICAR administration to accumulate FAT/CD36 on mitochondrial membranes, while acute treadmill running was performed on 25 week old male C57BL/6 WT and AMPK α2 kinase dead (KD) mice, originally produced by Dr. Birnbaum [26], to determine the necessity of AMPKα2 signaling on mitochondrial FAT/CD36 accumulation. All animals were bred onsite at the University of Guelph and housed in a climate- and temperature-controlled rooms on a 12:12-h light-dark cycle, with standard chow and water provided *ad libitum* (n = 4–8 per experiment). For all experiments, following anesthetization by an intraperitoneal injection of sodium pentobarbital (6 mg/kg) the experimental muscles were removed. This study was approved by the University of Guelph Animal Care Committee, and conforms to the guide for the care and use of laboratory animals published by the US National Institutes of Health.

### Electrical Stimulation-Induced Muscle Contraction

Rats were chosen instead of mice for these experiments for two reasons; 1) the control and contralateral stimulated muscles were taken from the same animal, and 2) each animal represented an independent experiment, both of which decreased the experimental variability compared to mice. Following anesthetization the femoral artery of the control limb was tied off, and the corresponding red gastrocnemius muscle excised. The sciatic nerve of the contralateral limb was exposed, and stimulating electrodes placed around the nerve as previously reported [10]. Electrical stimulation was applied for various durations (ie. 7.5, 15, 22.5 or 30 minutes) using a Grass stimulator with a train delivery of 100 Hz/3 s at 5 V, a train duration of 200 ms and a pulse duration of 10 ms. Following stimulation the red gastrocnemius muscle was excised and utilized to isolate sarcolemmal vesicles or mitochondria as described below.

### Isolation of Plasma Membranes

Giant sarcolemmal vesicles were prepared as previously described [10]. Briefly, muscle was cut into thin strips and incubated (1 hour, 34°C, 100 rpm) in 140 mM KCl/10mM MOPS (pH 7.4) containing collagenase VII (150 units/ml) and aprotinin (10 mg/ml). The muscle was washed with KCl/MOPS containing 10 mM Na_2_EDTA (pH 7.4) and the supernatants combined. Percoll (3.5%), KCl (28 mM) and aprotinin (10 μg/mL) were added to the supernatant. This solution was layered under 3 mL of 4% Nycodenz and 1 mL KCl/MOPS, and centrifuged (60 ×g, 45 min, 25°C). The vesicles were harvested from the interface of Nycodenz and KCl/MOPS and pelleted by centrifugation (9000 ×g, 10 min, 25°C) and the resulting pellet was resuspended in KCl/MOPS and stored at -80°C for Western blotting.

### Mitochondrial Isolation from Skeletal Muscle

Differential centrifugation was used to obtain both subsarcolemmal (SS) and intermyofibrillar (IMF) mitochondria from the red gastrocnemius (rats) or quadriceps (mice) muscles as described previously [27], with minor modifications. Briefly, muscle was minced and diluted in ice-cold mitochondrial isolation buffer (100 mM sucrose, 100 mM KCl, 50 mM Tris HCl, 1 mM KH_2_PO_4_, 0.1 mM EGTA, 1 mM ATP, 0.2% BSA; pH 7.4) and homogenized on ice with a tight fitting Teflon pestle at 750 rpm. The homogenate was centrifuged at 800 *g* for 10 min in order to separate the SS mitochondria from the myofibrils. The IMF mitochondria were resuspended in isolation buffer and treated with a protease (P5380; Sigma) for exactly 5 minutes and then centrifuged at 5000 *g* for 5 min to remove the myofibrils. Further centrifugation at 800 *g* for 10 min was used to recover the IMF mitochondria. The SS and IMF mitochondria were then spun twice at 10 000 *g* for 10 minutes, and the pellets resuspended in a standard respiration medium (120 mM KCl, 1 mM EGTA, 5 mM KH_2_PO_4_, 5 mM MgCl_2_ and 5 mM HEPES; pH 7.4). For Western blotting, the mitochondria were purified further by using a Percoll gradient (Sigma-Aldrich) as previously reported [28].

### Mitochondrial Bioenergetics

Rates of oxygen consumption were measured in isolated mitochondria (0.2 mg/ml) at 25°C by high-resolution respirometry in standard respiration medium (MiR05). The sequential respiration protocol consisted of determining state IV (leak respiration) respiration in the presence of 5 mM pyruvate + 2 mM malate, followed by state III respiration (coupled respiration) in the presence of 100 μM ADP to determine P/O ratios and finally 5 mM ADP to determine maximal state III (maximum phosphorylating) respiration. These values were used to determine P/O ratios and respiratory control ratios (RCR) as indexes of mitochondrial coupling to validate the mitochondrial isolation protocol.

### Western blot analysis

Muscle homogenates, purified isolated sarcolemmal vesicles and mitochondrial fractions were analyzed for total protein (BCA protein assay). Five micrograms of denatured protein from each sample were separated by electrophoresis on 7.5% and 12% SDS-polyacrylamide gels and transferred to a polyvinylidene difluoride membrane. Thereafter, membranes were blocked in 7.5% BSA and probed over night with commercially available antibodies against FAT/CD36 (rats; donated by Dr. Tandon; mice; Santa Cruz), FABPpm (donated by Dr. Calles-Escandon), COXIV (cytochrome c oxidase IV; Invitrogen), Cav-1 (caveolin-1; BD Biosciences), MCT1 (monocarboxylate transporter 1; Abcam), SERCA2 (sarcoplasmic/endoplasmic reticulum Ca^2+^-ATPase; Thermo Scientific), AMPKα total and AMPKα Thr172 phosphorylation (Cell Signaling), ACC (acetyl-CoA carboxylase) and ACC Ser79 phosphorylation (Cell Signaling), extracellular signal-regulated kinase (ERK1/2) total and Thr202/Tyr204 phosphorylation (Cell Signaling). All blots were quantified using enhanced chemiluminescence (Perkin Elmer, Woodbridge, ON) and quantified by densitometry (Alpha Innotech Fluorchem HD2, Fisher Scientific, Ottawa, ON).

### AICAR administration

Mice were randomly divided into two groups (WT saline (n = 6) or WT AICAR (n = 6)) and given a single intraperitoneal injection of saline (0.9% NaCl) or AICAR dissolved in saline (1 mg/g body weight). Tail blood was collected at time 0, 20 min and 60 min using a glucometer to assess the effectiveness of the AICAR injection before isolation of mitochondria. Animals were anesthetized with sodium pentobarbital (100 ul) and the quadriceps muscles rapidly removed after the 60 minute protocol.

### Acute exercise protocol

Before the exercise protocol, animals were familiarized to the treadmill at slow speeds (5–15 m min^-1^) for 5 consecutive days followed by a 48-hour rest before the start of the exercise bout. Acute exercise consisted of treadmill running for 60 minutes. Specifically, WT and AMPK KD mice ran at 12 m min^-1^ (5% incline), while WT mice also ran at 25 m min^-1^ (5% incline) to enable comparisons between absolute and relative intensities of exercise on mitochondrial FAT/CD36 accumulation. For signaling measurements, mice only ran for 30 minutes. Following cessation of exercise, the mice were immediately anesthetized and the quadriceps muscles harvested and immediately processed for mitochondrial extraction as outlined above.

### Statistics

All data were analyzed using t tests, except for the comparison of ERK1/2 phosphorylation following treadmill running at different intensities of exercise, which was analyzed using an ANOVA with a Fisher’s least significance difference post-hoc analyses. The data are presented as means ± S.E.M. Statistical significance was accepted at *P* < 0.05.

## Results

### Contraction-induced FAT/CD36 accumulation on sarcolemmal and mitochondrial membranes

**Fig 1:**
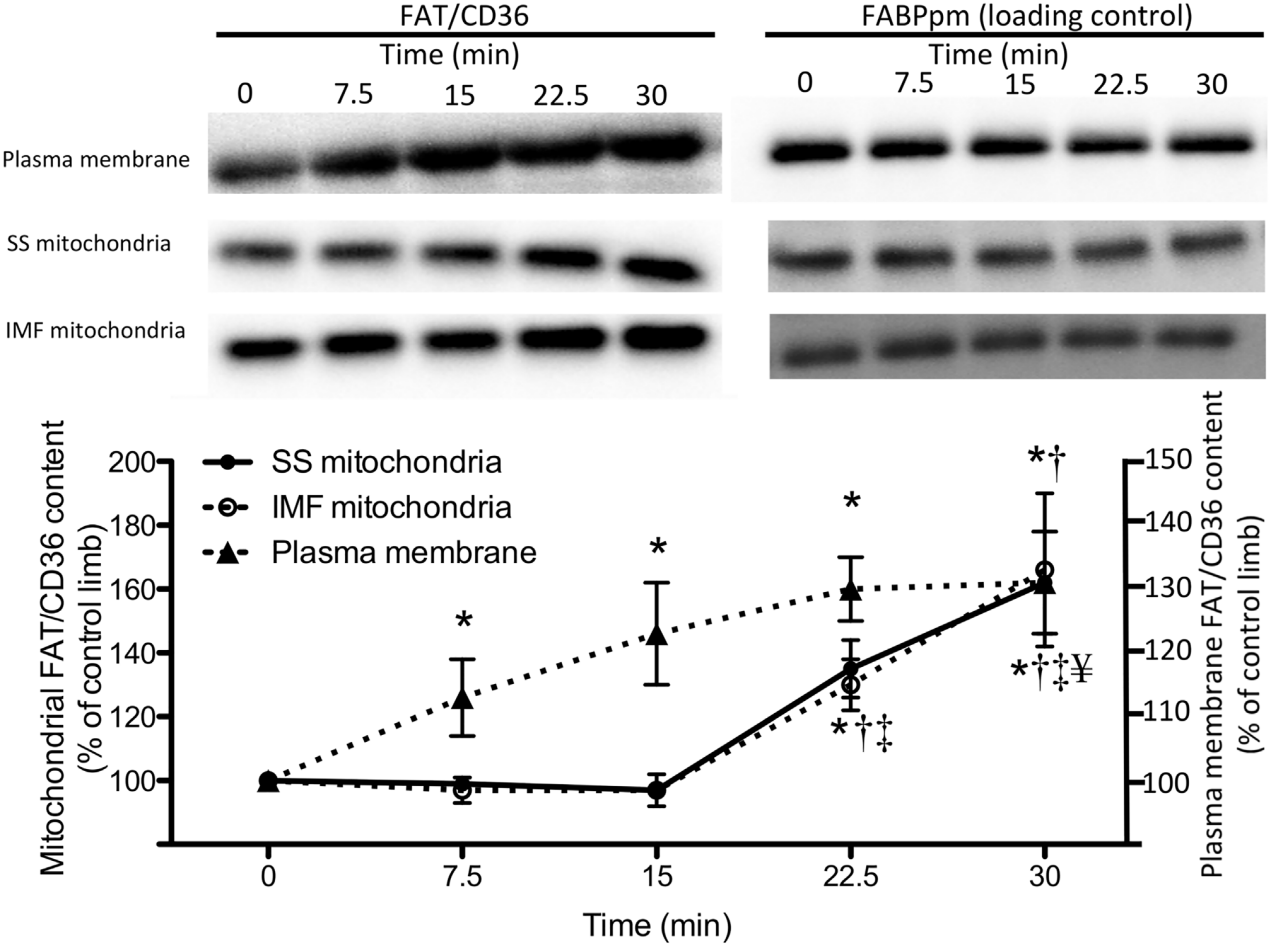
Temporal response of muscle contraction-induced FAT/CD36 accumulation on sarcolemmal, subsarcolemmal (SS) and intermyofibrillar (IMF) mitochondrial membranes. Sciatic nerve stimulation was used to determine FAT/CD36 accumulation, while plasma membrane fatty acid binding protein (FABPpm), which does not respond to contraction, was utilized as a control. While FAT/CD36 accumulated on the plasma membrane after 7.5 minutes, SS and IMF mitochondrial FAT/CD36 protein was not increased until 22.5 minutes. Values are reported as the means ± SEM (n = 7). * significantly (P<0.05) different from the contralateral control muscles (ie. time 0), † significantly (P<0.05) different from 7.5 minutes of stimulation, ‡ significantly (P<0.05) different from 15 minutes of stimulation, and ¥ significantly (P<0.05) different from 22.5 minutes of stimulation.

We first aimed to determine the contraction-induced time-course of FAT/CD36 accumulation on plasma and mitochondrial membranes, as a conserved signaling pathway would be expected to result in similar temporal responses between membranes. To this end we utilized sciatic nerve electrical stimulation in rats to invoke muscle contraction for various lengths of time and subsequently isolated sarcolemmal and mitochondrial membranes. These studies revealed unique temporal responses, as FAT/CD36 increased (P<0.05) on the plasma membrane after 7.5 minutes, and was further elevated following the 30-minute protocol (Fig 1).

While SS and IMF mitochondrial responses were identical, in contrast to the rapid responses observed at the plasma membrane, FAT/CD36 only increased (P<0.05) on mitochondrial membranes after 22.5 minutes of muscle contraction (Fig 1). In all membranes studied, FABPpm (as known as aspartate amino transferase in mitochondria [29]) did not change during the contraction protocol (Fig 1), nor did MCT1 on the plasma membrane or COXIV on mitochondrial membranes (data not shown). Therefore, the current data suggests a sequential response to muscle contraction, as FAT/CD36 accumulates on the plasma membrane rapidly while mitochondrial FAT/CD36 responses are delayed. These data support the notion that divergent signaling mechanisms may be responsible for inducing the accumulation of FAT/CD36 on plasma and mitochondrial membranes.

### Determining the role of AMPK signaling in mitochondrial FAT/CD36 accumulation

We next aimed to determine the signaling events responsible for mitochondrial FAT/CD36 accumulation during exercise. Studies in mice have previously shown that AMPKα2 is not required for inducing FAT/CD36 translocation to the plasma membrane [11], and therefore given the divergent temporal responses observed between plasma and mitochondrial membranes, we investigated the role of AMPK signaling in mitochondrial FAT/CD36 accumulation.

**Fig 2:**
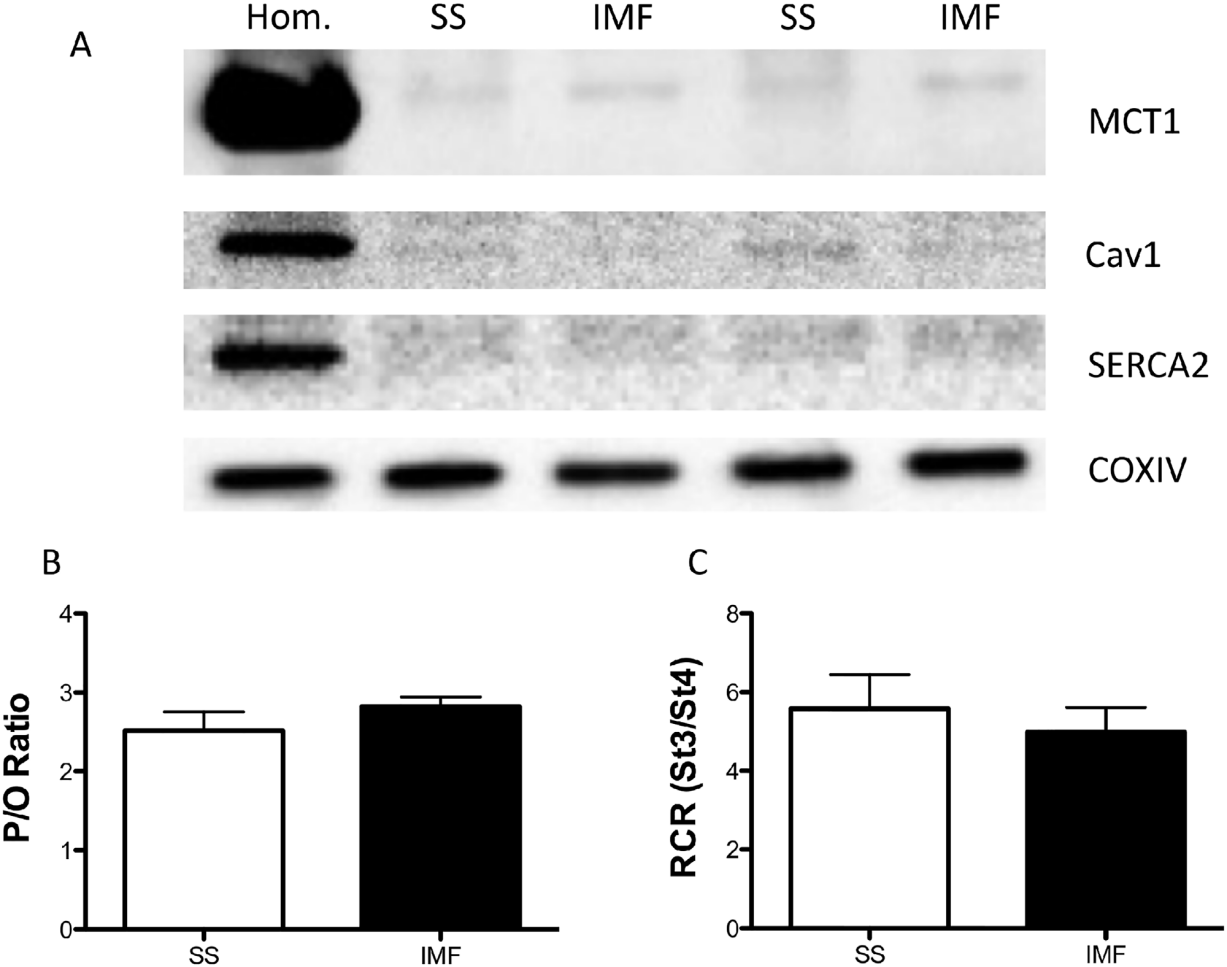
Representative Western blots on isolated subsarcolemmal (SS) and intermyofibrillar (IMF) mitochondria and assessment of mitochondrial respiratory coupling. Isolated mitochondria display an absence of MCT1, Cav1 and SERCA2, but the presence of COXIV, suggesting the preparation recovered mitochondria devoid of plasma membrane and sarcoplasmic reticulum contamination (A). Mitochondria were also utilized to assess coupling efficiencies as an index of mitochondrial damage. Specifically, the P/O ratios and RCR (reported in B) values suggest mitochondria were coupled, and therefore not damaged during the isolation procedure (B). Values are reported as the means ± SEM (n = 4).

Specifically, we aimed to determine if AICAR would induce mitochondrial FAT/CD36 accumulation and if the typical accumulation of mitochondrial FAT/CD36 observed during exercise would be prevented in AMPKα2 KD mice. The later experiments require the utilization of mice, and therefore we performed AICAR experiments in mice as well for consistency. However, we first confirmed the presence of mitochondria and the absence of alternative membrane contamination within our preparation. Specifically, we show the presence of COXIV in SS and IMF mitochondria, and the absence of MCT1, Cav-1 and SERCA2, suggesting neither the plasma membrane nor the sarcoplasmic reticulum contaminated our isolated mitochondria (Fig 2A). To further validate the mitochondrial isolation procedure we determined the coupling efficiency (P/O) and dynamic response to ADP (RCR). The average P/O ratios obtained from the SS and IMF mitochondria (Fig 2B) as well as the RCR from the SS and IMF mitochondria (Fig 2C) are comparable to literature values. Therefore, the mitochondria that were isolated were devoid of contamination and fully coupled.

### Effects of AICAR on skeletal muscle FAT/CD36 translocation to the SS and IMF mitochondria

**Fig 3:**
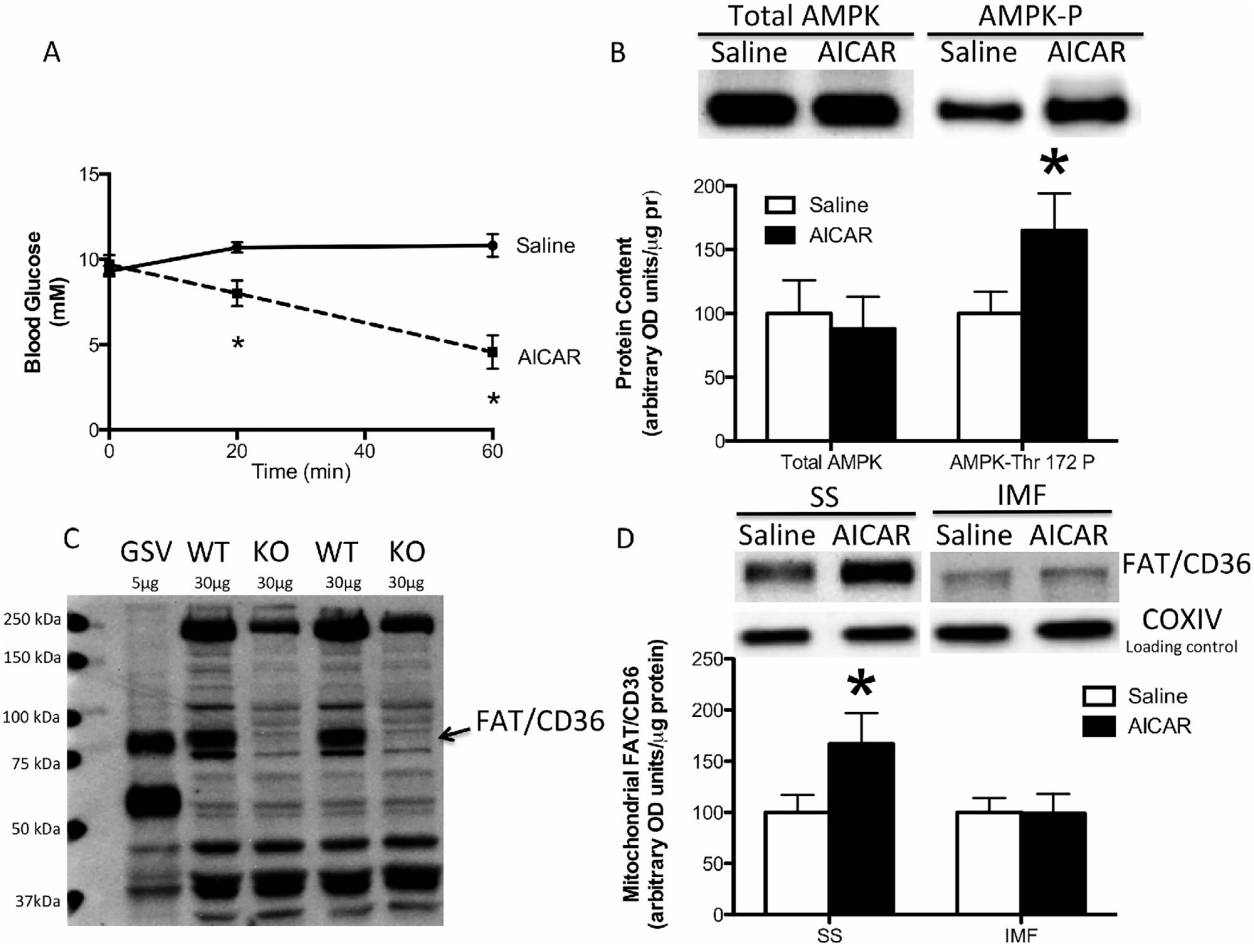
Effect of acute AICAR administration on mitochondrial FAT/CD36 content. An intraperitoneal injection of AICAR decreased blood glucose (A) and increased skeletal muscle AMPK Thr172 phosphorylation without altering total AMPK protein content (B). The FAT/CD36 antibody in mice is polyclonal, and therefore we confirmed that an ~88 kDa band was present in giant sarcolemmal vesicle (GSV) and mitochondrial samples from FAT/CD36 WT mice and absent in mitochondria isolated from FAT/CD36 knock out (KO) mice. A representative blot is provided (C). AICAR increased FAT/CD36 protein content in subsarcolemmal (SS) but not in intermyofibrillar (IMF) mitochondria. COXIV was utilized as a loading control. Muscle was taken 1 hour after the intraperitoneal injection of AICAR. Values are reported as the means ± SEM (n = 6). * significantly (P<0.05) different from saline.

To investigate the role of AMPK signaling in FAT/CD36 trafficking to mitochondrial membranes we first administered AICAR, a well-established AMPK agonist, to WT mice. Before isolating mitochondria, blood glucose measurements were taken to confirm the effectiveness of the AICAR injection. Blood glucose dropped by more than 50% with the AICAR injection while saline had no effect (Fig 3A). AICAR significantly (P<0.05) increased (+65%) the phosphorylation of AMPK-Thr172 in the quadriceps muscle without altering total AMPK protein (Fig 3B). Combined, these data validate AICAR-induced AMPK activation.

The FAT/CD36 antibody in mice is polyclonal, and therefore we confirmed that an ~88 kDa band was present in plasma membranes and mitochondrial samples from FAT/CD36 WT mice and absent in mitochondria isolated from FAT/CD36 knock out mice to verify its specificity (Fig 3C). The expected ~88 kDa band for FAT/CD36 was clearly present in plasma membranes, and in the FAT/CD36 WT mice (Fig 3C). In contrast, the 88 kDa band was lost in the FAT/CD36 knock out mice, confirming the polyclonal FAT/CD36 antibody. Therefore, we next examined the impact of AICAR on mitochondrial FAT/CD36 content. In WT mice, AICAR significantly (P<0.05) increased FAT/CD36 content in SS mitochondria from the quadriceps muscle (+67%) but had no effect on IMF mitochondria (Fig 3D). These data suggest that AMPK signaling can induce mitochondrial FAT/CD36 accumulation, at least within SS mitochondria.

### The necessity of AMPK for mitochondrial FAT/CD36 accumulation

**Fig 4:**
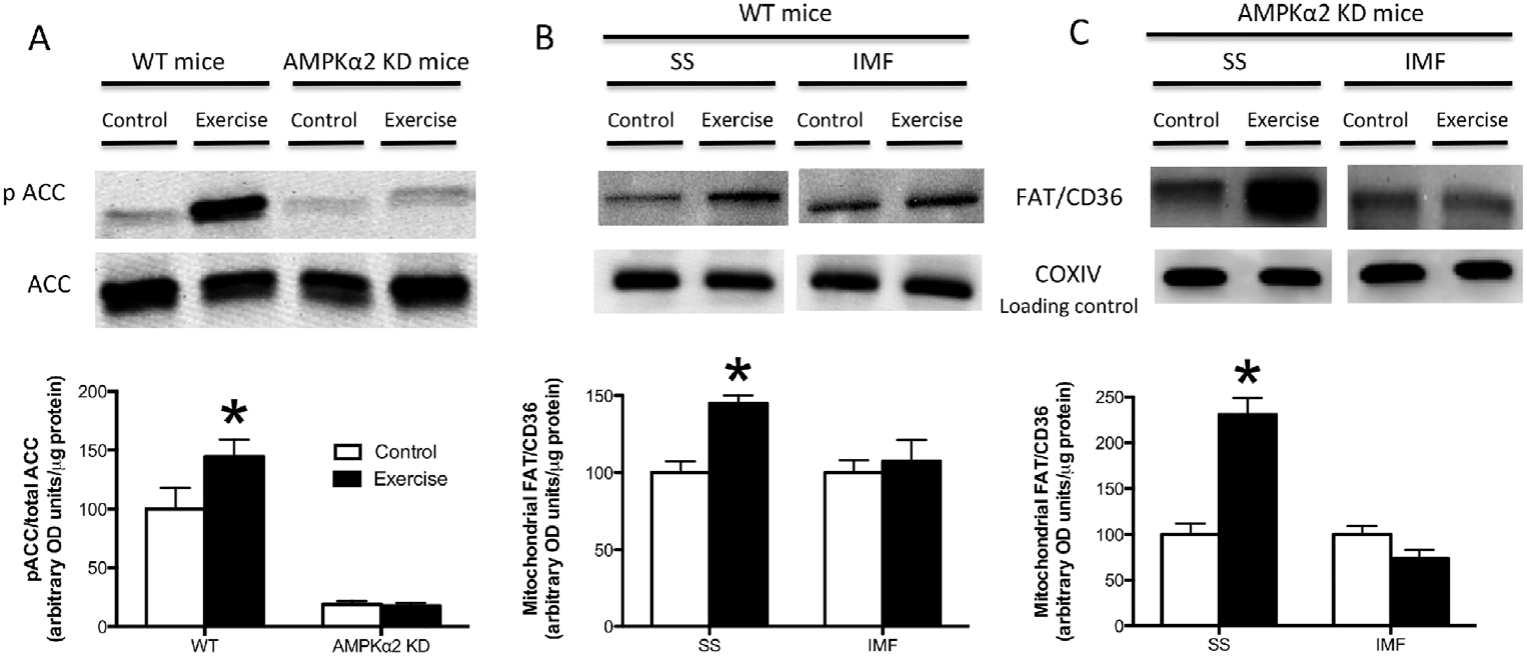
Effects of acute treadmill running on FAT/CD36 mitochondrial content in WT and AMPKα2 KD mice. ACC Ser79 phosphorylation was examined in whole muscle (A), while subsarcolemmal (SS) and intermyofibrillar (IMF) mitochondria were isolated from WT (B) and AMPKα2 KD (C) mice run at 12m/min at a 5% grade for an hour or from sedentary controls. Mitochondria were utilized to determine FAT/CD36 protein content, while COXIV was utilized as a loading control. Values are reported as the means ± SEM (n = 4 for ACC Westerns, n = 8 for mitochondrial Westerns). *significantly (P<0.05) different from control.

**Fig 5:**
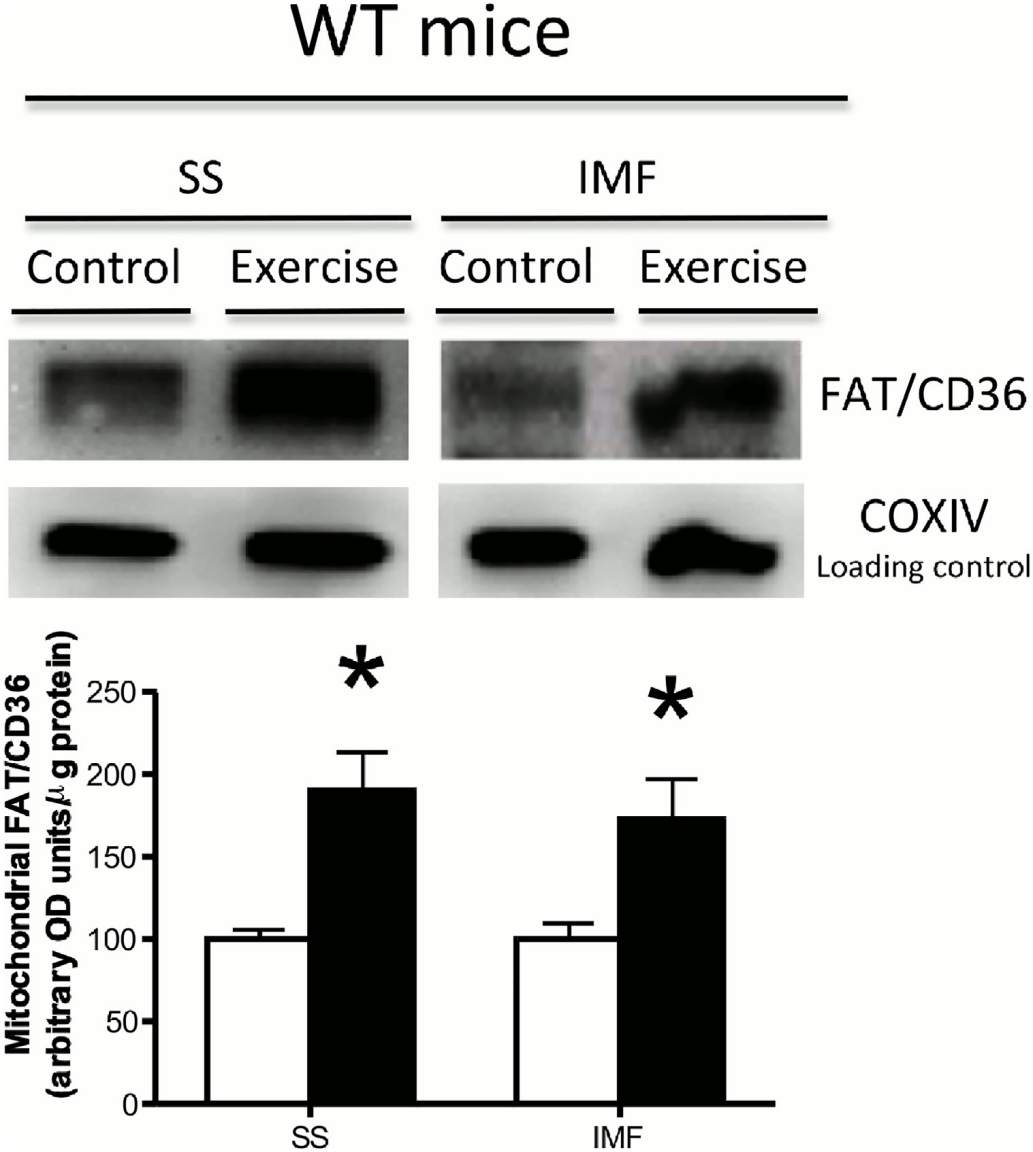
Effects of acute treadmill running to exhaustion on FAT/CD36 mitochondrial content in WT mice. Subsarcolemmal (SS) and intermyofibrillar (IMF) mitochondria were isolated from WT (A) mice run at 25 m/min at a 5% grade for an hour or from sedentary controls. Mitochondria were utilized to determine FAT/CD36 protein content, while COXIV was utilized as a loading control. Values are reported as the means ± SEM (n = 6). *significantly (P<0.05) different from control.

**Fig 6:**
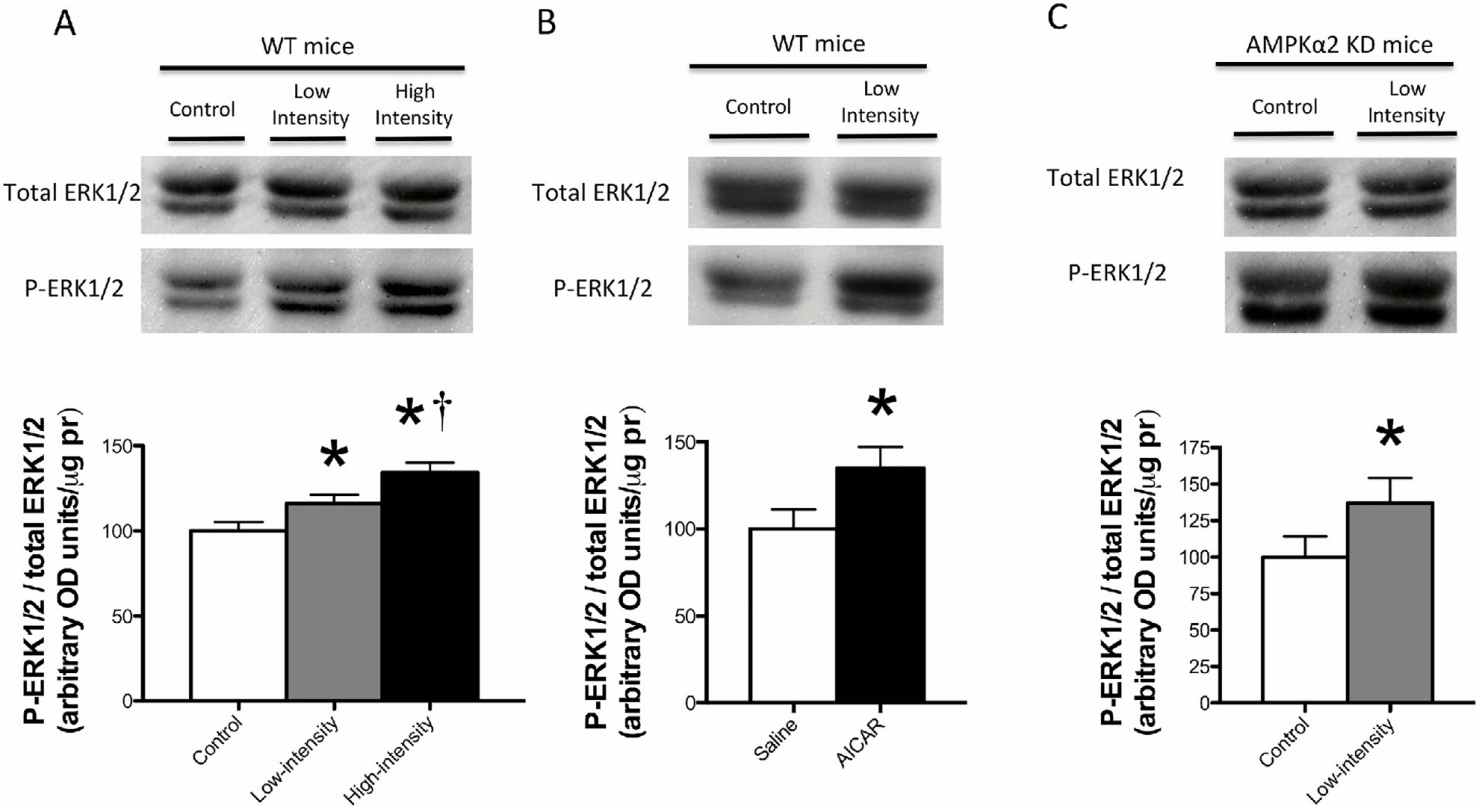
Effects of AICAR and acute treadmill running on ERK1/2 phosphorylation. Treadmill running for 30 minutes increased ERK1/2 Thr202/Tyr204 phosphorylation in an intensity-dependent manner in WT mice (A). ERK1/2 Thr202/Tyr204 phosphorylation increased 30 minutes following an intraperitoneal injection of AICAR in the absence of alterations in total protein content in WT mice (B). Treadmill running for 30 minutes increased ERK1/2 Thr202/Tyr204 phosphorylation in AMPKα2 KD mice (C). Values are reported as the means ± SEM (n = 5). * significantly (P<0.05) different from control (saline or sedentary) and † significantly (P<0.05) different from low-intensity running.

Given that AICAR induced FAT/CD36 accumulation within SS mitochondria, we next utilized transgenic mice that express a kinase dead (KD) α2 AMPK isoform to determine if AMPK was required for FAT/CD36 translocation to the mitochondria during an acute bout of exercise. In the quadriceps muscle, acute treadmill running at 12 m/min increased ACC Ser79 phosphorylation in WT mice, but not in AMPKα2 KD mice (Fig 4A), confirming the phenotype of the animals. Treadmill running increased FAT/CD36 protein on SS mitochondrial membranes in both WT and AMPKα2 KD mice (Fig 4B and 4C). In contrast, regardless of genotype FAT/CD36 was not increased in IMF mitochondria (Fig 4B and 4C). These data suggest that AMPKα2 is not required for FAT/CD36 translocation to the SS mitochondria. However, it was striking that while severe muscle contraction (electrical stimulation) induced FAT/CD36 accumulation in both SS and IMF mitochondria (Fig 1), neither AICAR (Fig 3) nor treadmill running at 12 m/min induced FAT/CD36 translocation to IMF mitochondria (Fig 4). These data may suggest that exercise intensity influences IMF mitochondrial FAT/CD36 protein accumulation. AMPKα2 KD mice are known to have exercise-intolerance [30]. As a result, in the current study the treadmill speed required to ensure AMPKα2 KD animals ran for 60 minutes was very low. Therefore, to determine if the absence of IMF mitochondrial FAT/CD36 accumulation was intensity dependent, we ran a separate group of WT animals at a much higher treadmill speed (25 m/min). In these mice, FAT/CD36 protein was increased ~2-fold on both SS and IMF mitochondria (Fig 5), suggesting FAT/CD36 accumulation on mitochondrial membranes is exercise intensity dependent, similar to observations at the plasma membrane [9]. Interestingly, the magnitude of increase in SS mitochondrial FAT/CD36 was similar between WT and AMPKα2 KD mice that were essentially run to exhaustion, however IMF mitochondrial FAT/CD36 only increased in WT mice (Figs 4 and 5). These data may suggest that AMPKα2 is required for IMF mitochondrial accumulation, or alternatively that IMF FAT/CD36 accumulation is regulated by absolute, and not relative intensity of exercise. To further examine alternative signaling that could regulate IMF mitochondrial FAT/CD36 accumulation, we examined ERK1/2. We show in WT mice that ERK1/2 phosphorylation is exercise-intensity dependent (Fig 6A), similar to the observed accumulation of mitochondrial FAT/CD36 (Figs 4 and 5), suggesting ERK1/2 may mediate IMF mitochondrial FAT/CD36 translocation. However, in contrast, AICAR induced ERK1/2 phosphorylation (Fig 6B), and ERK1/2 phosphorylation was similarly induced with exercise in AMPKα2 KD mice (Fig 6C), both of which occurred in the absence of IMF mitochondrial FAT/CD36 accumulation (Figs 3D and 4B). Therefore, while it remains possible that ERK1/2 contributes to SS mitochondrial FAT/CD36 accumulation, ERK1/2 clearly does not mediate IMF mitochondrial FAT/CD36 accumulation.

## Discussion

In the current study we provide evidence that 1) FAT/CD36 rapidly accumulates on sarcolemmal membranes at the onset of muscle contraction, while mitochondrial FAT/CD36 accumulation is delayed, 2) AICAR induces SS mitochondrial FAT/CD36 accumulation, but 3) exercise-induced accumulation of SS mitochondrial FAT/CD36 protein is not altered in AMPKα2 KD mice. In addition, we show that 4) FAT/CD36 accumulation on mitochondria is exercise intensity dependent, and 5) appears to be associated with ERK1/2 phosphorylation. Combined, these data suggest that activation of AMPK signaling is sufficient to induce mitochondrial FAT/CD36 trafficking events, but highlight that AMPKα2-independent signaling mechanisms influence mitochondrial FAT/CD36 translocation during muscle contraction.

### Rapid responses in sarcolemmal FAT/CD36 accumulation during muscle contraction

The redistribution of FAT/CD36 to the plasma membrane appears to be a major regulator of fatty acid oxidation during exercise, as ablating FAT/CD36 severely compromises exercise tolerance while increasing rates of glycogen breakdown [8]. In the current study we provide evidence that this response occurs rapidly to muscle contraction, as sarcolemmal FAT/CD36 protein was increased after 7.5 minutes of muscle contraction. Previous research in rodents has shown that 1 minute of muscle contraction is sufficient to induce plasma membrane FAT/CD36 accumulation, which was not further stimulated with continued muscle contraction (10 minutes) [11]. However, the current data suggests that while contraction-induced FAT/CD36 accumulation on the plasma membrane is rapid, only ~50% of the response occurred during the first 7.5 minutes of muscle contraction, as 30 minutes of contraction increased sarcolemmal FAT/CD36 to a greater degree. These data suggest a temporal component to exercise-induced sarcolemmal FAT/CD36 accumulation. In humans, 2 hours of cycling at ~65% VO_2peak_ increased FAT/CD36 protein ~50% on the plasma membrane [31], however early time points during exercise have not been examined, and therefore the temporal response in humans remains unknown. The response of sarcolemmal FAT/CD36 accumulation in the current study mirrors the known exercise-induced increases in fatty acid oxidation [32, 33], suggesting translocation of FAT/CD36 to the plasma membrane contributes to the well-known ability of fatty acid availability in muscle to influence fuel selection during exercise.

### Delayed responses in mitochondrial FAT/CD36 accumulation during muscle contraction

In contrast to the rapid response at the plasma membrane, FAT/CD36 accumulation on mitochondrial membranes appears to be delayed, as an increase in SS and IMF mitochondrial FAT/CD36 was only observed after 22.5 minutes of muscle contraction. Therefore, it appears that initial molecular responses during muscle contraction involve stimulating plasma membrane fatty acid transport, and subsequently at the mitochondrial membrane. The current data therefore shows that mitochondrial FAT/CD36 accumulation displays a temporal delay at the onset of exercise. This is supported by findings in humans, which have shown that exercise induces FAT/CD36 accumulation on mitochondrial membranes following 2 hours of cycling at ~65% VO_2peak_, but not after 30 minutes of cycling [34]. The lack of FAT/CD36 accumulation on mitochondrial membranes in humans after 30 minutes of exercise likely reflects the intensity of muscle contraction, as the 30 minutes of electrical stimulation used in the current study is much more intense than moderate-intensity cycling. In addition, the current data indicates that FAT/CD36 only accumulates on SS mitochondria during low-intensity exercise, and the human experiments pooled SS and IMF mitochondria [34], which may further explain the previously reported lack of a response in mitochondrial FAT/CD36 during the onset of low-intensity exercise in humans [34]. Altogether, the current data shows that signals initiated during muscle contraction result in divergent temporal responses in plasma and mitochondrial membrane FAT/CD36 accumulation.

### The role of AMPK in mediating mitochondrial FAT/CD36 accumulation

In the current study, AICAR administration resulted in SS mitochondrial FAT/CD36 accumulation, suggesting AMPK is sufficient to induce mitochondrial FAT/CD36 accumulation. However, SS mitochondrial FAT/CD36 protein accumulation was not prevented in AMPKα2 KD mice following an acute treadmill run, indicating AMPKα2 is not required in these molecular events. These observations are remarkably consistent with the previously described role for AMPK in mediating FAT/CD36 translocation to the plasma membrane, where it has also been suggested to be sufficient but not required [11]. Combined these data suggest that redundant signaling cascades regulate mitochondrial FAT/CD36 trafficking in muscle during exercise. It is possible that the decrease in blood glucose concentrations observed during the AICAR experiment activated signaling pathways independent of AMPK that regulate mitochondrial FAT/CD36 accumulation. This may partially explain why SS mitochondrial FAT/CD36 accumulation was not affected in AMPKα2 KD mice. One potential alternative signaling pathway is ERK1/2, as the provision of PD-98059, a purported ERK1/2 specific inhibitor, has previously been shown to prevent sarcolemmal FAT/CD36 translocation and fatty acid transport into contracting muscle, suggesting ERK1/2 is required for FAT/CD36 sarcolemmal trafficking [12]. The current study provides evidence that ERK1/2 signaling may also be involved in mediating SS mitochondrial FAT/CD36 trafficking. Specifically, this suggestion is supported by the observation that both ERK1/2 phosphorylation and SS mitochondrial FAT/CD36 protein are exercise-intensity dependent, and AICAR increased ERK1/2 phosphorylation, similar to a previous report [35]. However, these results are associative and do not indicate a cause-and-effect relationship between ERK1/2 and mitochondrial FAT/CD36 trafficking. In addition, ERK1/2 phosphorylation was increased similarly in WT and AMPKα2 KD mice (~35%), however only WT mice displayed an accumulation of IMF mitochondrial FAT/CD36, suggesting additional mechanisms contribute to the subcellular location of FAT/CD36. Moreover, if ERK1/2 is involved in regulating both sarcolemmal and mitochondrial FAT/CD36 accumulation, a mechanism explaining the divergent temporal responses between these biological membranes during exercise remains ambiguous. Nevertheless, the present study provides evidence that AMPKα2 is not required in these events.

## Perspectives and Limitations

AMPK is a heterotrimeric protein that is composed of α catalytic, β scaffolding and nucleotide-binding γ subunits (reviewed in [36]). While several isoforms of the heterotrimeric AMPK protein exist, isoforms containing AMPKα2 are thought to represent the more prominent catalytic activity during steady state exercise/low intensity exercise [37, 38], when fatty acid oxidation is optimal [39]. It is for this reason that the transgenic mice utilized in the current study contained an inactive α2 subunit. However, these animals still possess AMPKα1 catalytic activity [11, 26, 30], and as a result skeletal muscle palmitate oxidation is not compromised [11, 26, 30]. In contrast, mice lacking the primary upstream AMPK kinase serine/threonine kinase 11 (LKB1) [40, 41] or global reduction in both AMPKα1 and 2 isoforms display impairments in fatty acid oxidation [42], suggestion complete ablation of AMPK signaling is required to compromise fatty acid oxidation. Therefore, future work should determine if mitochondrial FAT/CD36 accumulation during exercise is prevented when AMPK signaling is completely compromised to solidify the influence of AMPK signaling in mediating mitochondrial FAT/CD36 protein and fatty acid oxidation rates. The current finding that mitochondrial FAT/CD36 accumulation is exercise-intensity dependent could support the supposition that this protein regulates mitochondrial ATP provision during exercise. While the functional role of mitochondrial FAT/CD36 remains uncertain [17, 19, 43], we have previously provided evidence to suggest that FAT/CD36 interacts with acyl-CoA synthetase to affect rates of fatty acid oxidation, as ablation of FAT/CD36 reduces palmitate, but not palmitoyl-CoA, supported respiration [17]. These data are supported by the finding that in L6E9 myotubes overexpression of FAT/CD36 increases mitochondrial palmitate oxidation rates [20]. These data may also partially explain the known exercise intolerance of FAT/CD36 knock out mice, and the established increased reliance on carbohydrates during exercise in these animals [8]. However, it is difficult to delineate the independent contribution of attenuated plasma membrane transport and mitochondrial fatty acid oxidation in the exercise responses observed in these animals. Nevertheless, while not a uniform finding [43], there is now considerable evidence to support the presence of FAT/CD36 on mitochondrial membranes, as seven independent laboratories have reported FAT/CD36 on mitochondrial membranes in several tissues including skeletal muscle, liver and heart in a variety of species (rat, mouse, humans and cells) [15, 17–21, 44]. Regardless of these discrepancies within the literature and limitations in the genetic mice utilized, the current data provides evidence that AMPKα2 is not required in mediating mitochondrial FAT/CD36 trafficking.
